# Spatial co-location patterns between early COVID-19 risk and urban facilities: a case study of Wuhan, China

**DOI:** 10.3389/fpubh.2023.1293888

**Published:** 2024-01-04

**Authors:** Guoqing Zhi, Bin Meng, Hui Lin, Xin Zhang, Min Xu, Siyu Chen, Juan Wang

**Affiliations:** ^1^Electronic Science Research Institute of China Electronics Technology Group Corporation, Beijing, China; ^2^National Engineering Laboratory for Public Security Risk Perception and Control by Big Data, Beijing, China; ^3^College of Applied Arts and Sciences, Beijing Union University, Beijing, China; ^4^Laboratory of Urban Cultural Sensing & Computing, Beijing Union University, Beijing, China; ^5^Aerospace Information Research Institute, Chinese Academy of Sciences, Beijing, China; ^6^Southwest United University Campus, Yunnan Normal University, Kunming, China; ^7^The Engineering Research Center of GIS Technology in Western China of Ministry of Education of China, Kunming, China

**Keywords:** COVID-19, spatial risk, co-location, urban facilities, Geodetector, Wuhan, China

## Abstract

**Introduction:**

COVID-19, being a new type of infectious disease, holds significant implications for scientific prevention and control to understand its spatiotemporal transmission process. This study examines the diverse spatial patterns of COVID-19 within Wuhan by analyzing early case data alongside urban infrastructure information.

**Methods:**

Through co-location analysis, we assess both local and global spatial risks linked to the epidemic. In addition, we use the Geodetector, identifying facilities displaying unique spatial risk characteristics, revealing factors contributing to heightened risk.

**Results:**

Our findings unveil a noticeable spatial distribution of COVID-19 in the city, notably influenced by road networks and functional zones. Higher risk levels are observed in the central city compared to its outskirts. Specific facilities such as parking, residence, ATM, bank, entertainment, and hospital consistently exhibit connections with COVID-19 case sites. Conversely, facilities like subway station, dessert restaurant, and movie theater display a stronger association with case sites as distance increases, hinting at their potential as outbreak focal points.

**Discussion:**

Despite our success in containing the recent COVID-19 outbreak, uncertainties persist regarding its origin and initial spread. Some experts caution that with increased human activity, similar outbreaks might become more frequent. This research provides a comprehensive analytical framework centered on urban facilities, contributing quantitatively to understanding their impact on the spatial risks linked with COVID-19 outbreaks. It enriches our understanding of the interconnectedness between urban facility distribution and transportation flow, affirming and refining the distance decay law governing infectious disease risks. Furthermore, the study offers practical guidance for post-epidemic urban planning, promoting the development of safer urban environments resilient to epidemics. It equips government bodies with a reliable quantitative analysis method for more accurately predicting and assessing infectious disease risks. In conclusion, this study furnishes both theoretical and empirical support for tailoring distinct strategies to prevent and control COVID-19 epidemics.

## 1 Introduction

In late 2019, an unidentified pneumonia outbreak emerged in Wuhan, Hubei Province, China, later termed COVID-19 by the World Health Organization (WHO). This event captured global attention, and in the following months, the COVID-19 outbreak spread rapidly worldwide, originating in Wuhan and posing significant challenges. By early March 2020, the WHO officially designated it as a global pandemic (1). Governments implemented preventive measures to curb its spread, but the varied characteristics of urban infrastructure and differences in population density created challenges for authorities in gathering quantitative evidence of spatial patterns and formulating more targeted policies (2). Decision-makers were tasked with assessing the impact of these facilities on virus transmission risk, determining whether natural and socioeconomic factors influenced the severity of the outbreak, and understanding their role in urban virus propagation (3–6). Which factors significantly contribute to spatial risk discrepancies in outbreaks (7–9)?. What factors warrant comprehensive consideration for effective epidemic control (10, 11)?. This study aims to pinpoint variations in epidemic spread risk linked to various types of urban facilities to enhance public health protection. While there have been advancements in controlling the overall COVID-19 pandemic, the specific origins of this novel infectious disease outbreak and the mechanisms driving its rapid initial dissemination remain inconclusive (12, 13). Experts suggest that as human activity increases, the likelihood of similar infectious disease outbreaks persists. Although respiratory diseases share similarities in transmission, understanding the precise transmission dynamics of each major outbreak, especially in their early unnoticed stages, proves vital for early detection and control. Thus, this research zeroes in on the spatiotemporal transmission factors during the specific initial phase of this epidemic outbreak, carrying significant academic and practical importance.

In the strategy of effectively responding to the COVID-19 epidemic, the integration of multi-source big data and the use of geographic information systems (GIS) have become a new trend (14). Many scholars have used GIS and spatial analysis methods to study various aspects, such as natural environment (3), population factors (6), urban facilities (15), and human behavior (16), showing diverse features in data and method usage. At the level of natural environment, some studies have found that natural factors have a significant impact on COVID-19 risk (17, 18). For example, under the same natural environmental factors, the number of COVID-19 cases shows a certain correlation. Specifically, under the condition of controlling population migration, weather conditions with low temperature, small diurnal temperature range, and low humidity are more conducive to the spread of the epidemic (17). In terms of urban spatial factors, existing research has used hotspot analysis to explore COVID-19 high-risk areas, focusing on possible outbreak points in the city (19). Furthermore, various regression models have been used to analyze the main factors of COVID-19 epidemic aggregation (20). However, in large cities, the factors influencing COVID-19 spatial risks become more complex, and a single influencing factor appears inadequate in attribution analysis. It is necessary to combine multi-source socio-economic data and analyze the relationship between COVID-19 epidemic spatial distribution characteristics and urban geometric morphology and population social structure (21). At the individual level, previous studies have used big data to obtain people’s spatiotemporal behavior and explore the spatial risks of the epidemic. For example, using social media data to explore the spreading characteristics of the early COVID-19 epidemic in Wuhan, China (22); based on individual spatiotemporal behavior and epidemic risk. Using multi-level Bayesian models to quantify the probability of a person being infected on a train based on their seating position and riding time and interval (23). The above scholars have studied the development and risk of COVID-19 epidemic in time and space from multiple perspectives, and by integrating multi-source data and various spatial analysis methods, the research results are more guiding and scientific.

However, COVID-19 is more likely to spread within highly populated cities, as people stay for different lengths of time in different types of facilities (e.g., longer in hotels and shorter in bus stations), facilities have different levels of enclosure (e.g., hotels are enclosed spaces while bus stations are open spaces), and people have different protective measures in different city facilities (e.g., removing masks to eat in restaurants but wearing masks in crowded subway stations). This leads to varying levels of risk for people to be infected with coronavirus 2019-nCoV when engaging in activities in different types of facilities in the city. However, there is limited research quantifying the risk of infection from these facilities. In fact, there are many reasons why people may become infected in these facilities and places. It is necessary to know the distance from these facilities that can greatly reduce the risk of infection, and this is a prerequisite for exploring the factors that contribute to the risk of infection from different facilities in the context of COVID-19. Although COVID-19 has been ongoing for 3 years, current research results cannot well explain the following three issues: (1) what are the differences in COVID-19 epidemic risk among different city facilities, (2) what is the maximum distance that these facilities can make people infected with COVID-19, and (3) whether there are other factors that work together with some facilities to explain the spatial risk patterns of COVID-19 infection.

This research use the multiscale co-location method, exploring the spatial patterns between the early COVID-19 epidemic in Wuhan, Hubei Province, China, and urban facilities, calculates the epidemic risk distance of different facilities, and then quantitatively analyzes and tests the coordination of key facilities, discovering the hidden features of the COVID-19 epidemic in spatial diffusion in Wuhan. The research results have two practical implications: (1) they can guide relevant infectious disease departments to pay attention to facilities and factors that pose high transmission risks in cities, optimize the allocation of infectious disease prevention and control resources; (2) in the post-epidemic era, when similar infectious diseases occur, different attitudes can be taken towards the risks of different types of facilities in the city to avoid large-scale indiscriminate prevention and control. In terms of theoretical methods, this study uses a global scale coordination location analysis to screen facilities with more obvious spatial coordination patterns for in-depth discussion, and combines other natural and socio-economic factors to analyze the interaction of facility types with unknown coordination influence to discover the spatial risk heterogeneity of COVID-19 caused by multiple factors. This study is divided into three parts: the study area, study data, and research methods will be introduced in Part 2; the research results will be discussed in Part 3; and the research conclusions will be discussed in Part 4.

## 2 Materials and methods

### 2.1 Study area

**Figure 1:**
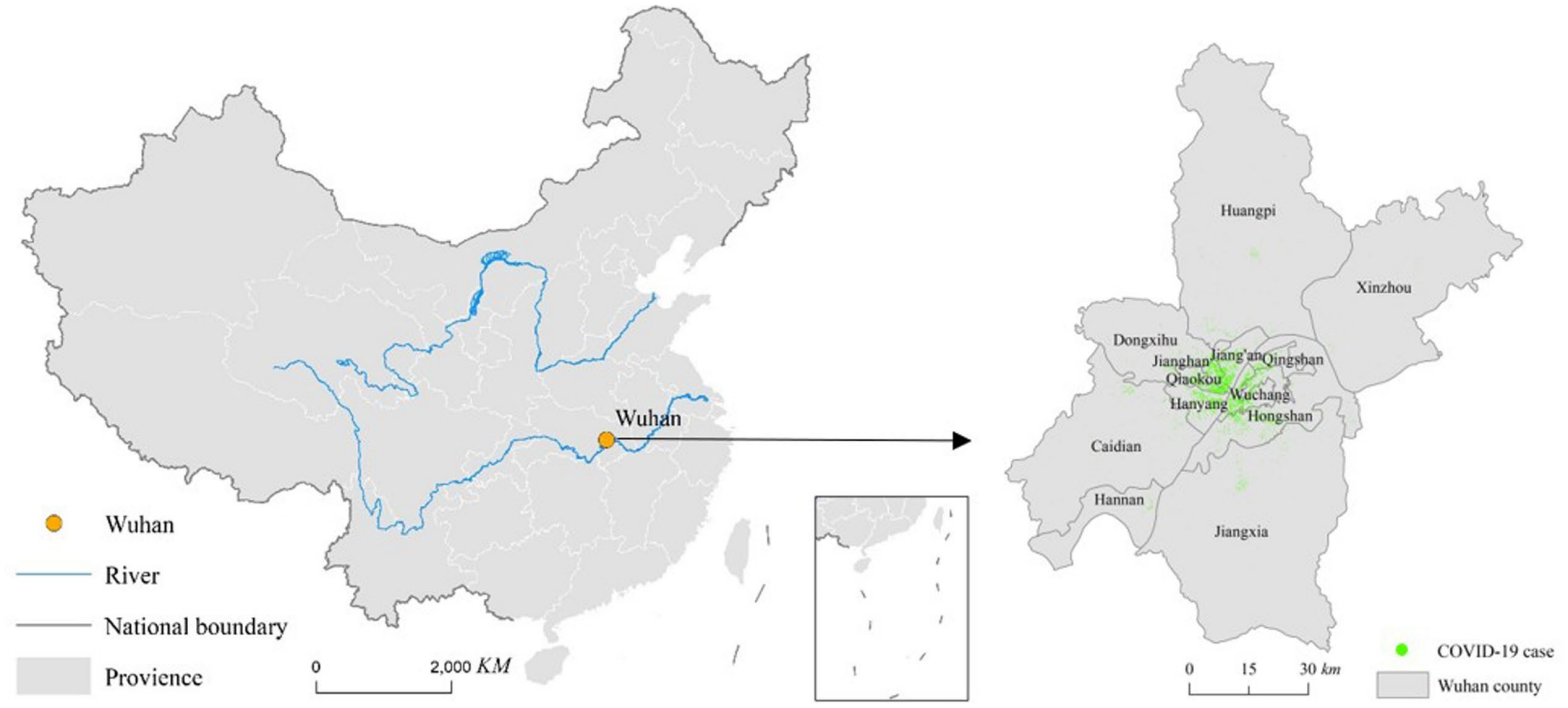
Study area.

Wuhan is located in central China, at the confluence of the Yangtze River and the Han River. Wuhan has a subtropical monsoon climate, with hot and rainy summers and mild and humid winters. Wuhan is the capital of Hubei Province and the city with the largest population in Hubei Province. As Wuhan is located in the Jianghan Plain, it is the largest comprehensive transportation hub for river, railway, and air transportation in the inland regions of China. The high-speed railway network covering almost half of China passes through Wuhan, and its airport provides direct flights to the five continents of the world. Therefore, the flow of people and goods is very frequent, leading to the rapid spread of COVID-19 to China and the world. The administrative divisions of Wuhan include the central area and the suburbs. The central area includes Jiang’an District, Jianghan District, Qiaokou District, Hanyang District, Wuchang District, Qingshan District, and Hongshan District. The suburbs include Dongxihu District, Hannan District, Caidian District, Jiangxia District, Huangpi District, and Xinzhou District (Figure 1).

### 2.2 Data sources

#### 2.2.1 COVID-19 cases data

Through our efforts, we have obtained COVID-19 case data used in the research, which comes from confirmed cases by the Chinese Center for Disease Control and Prevention (CDC). This database not only records the diagnosis time of cases, but also conducts retrospective investigations on the onset date of cases, which is more important for studying the temporal and spatial process of the disease. Therefore, when selecting cases, we all use the onset time, which is relatively more accurate in reflecting the temporal sequence of the disease compared to the newly diagnosed cases announced daily by the Wuhan municipal government. This data has been de-identified and obtained permission from the relevant responsible units. The onset date of the case data for this study starts from the onset date of the first confirmed case in China, which was at 0:00 on December 2, 2019, until the day before Wuhan implemented the close policy, which was at 24:00 on January 22, 2020. This data set includes 8,929 case data, and the main fields included in each case include the onset date, diagnosis time, reporting unit, home address, and population classification, which provide a good foundation for our more accurate spatial analysis.

#### 2.2.2 Urban facility data

The facility POI points used in this study are all from the download platform opened by Gaode (https://lbs.amap.com/), with main fields including facility name, major category, subcategory, latitude and longitude coordinates, and administrative region. Recent studies have shown a correlation between the spatial distribution of COVID-19 cases and facility distribution. Therefore, this study selected 27 facilities based on factors that may contribute to the occurrence of clustered COVID-19 outbreaks, and analyzed them separately.

#### 2.2.3 Remote sensing datasets

**Figure 2:**
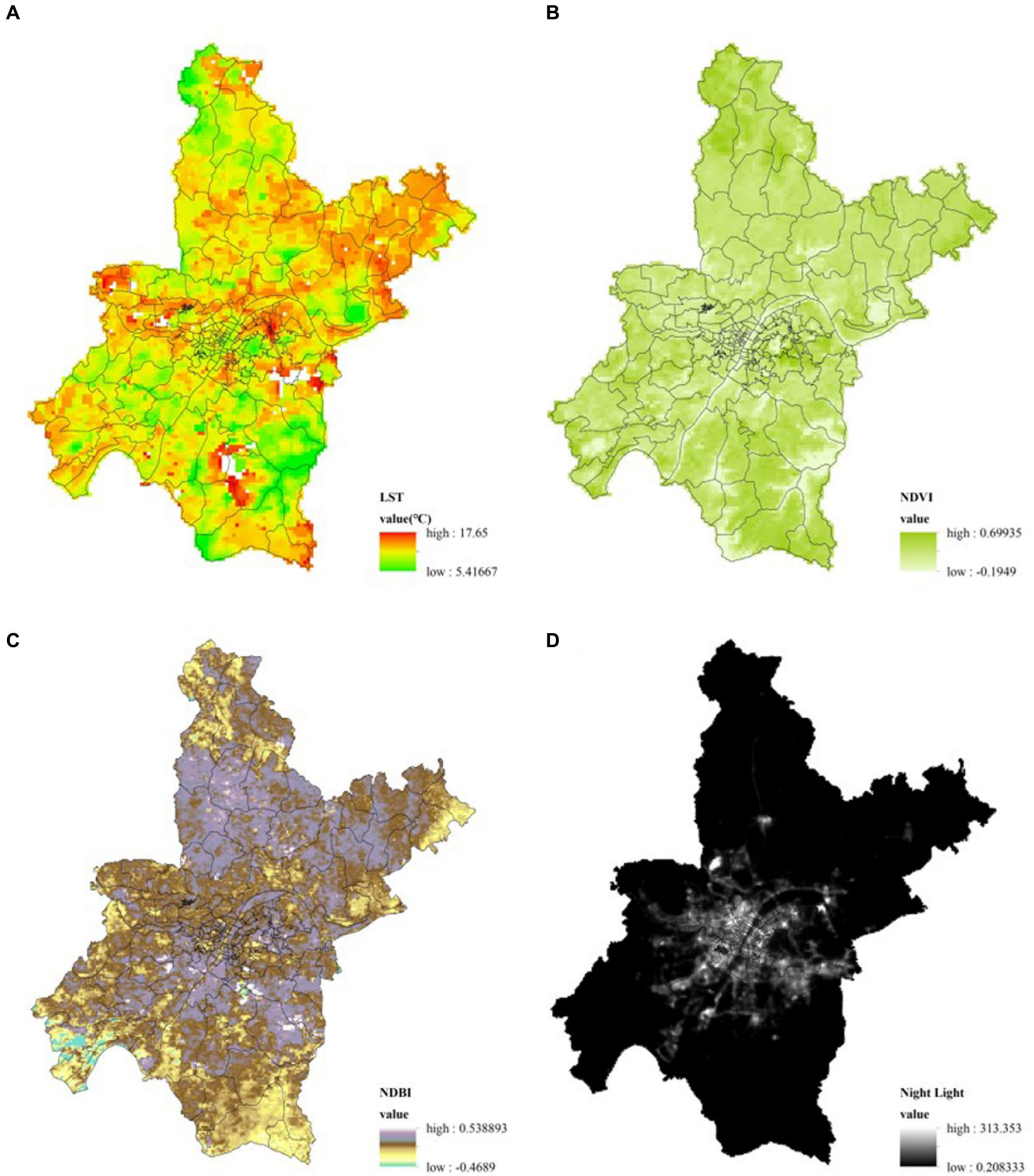
Remote sensing image map of Wuhan from Jan 1th to Jan 22nd, 2020 (averaging). **(A)** Land surface temperature; **(B)** NDVI; **(C)** NDBI; **(D)** Night light.

The remote sensing data used in this study are divided into four types: land surface temperature (LST), Normalized Difference Vegetation Index (NDVI), Normalized Difference Building Index (NDBI), and Night Light Index (NLI). This study selected images from January 1, 2020 to January 22, 2020 for normalization, mosaicking, and clipping, resulting in normalized image data for Wuhan from January 1, 2020 to January 22, 2020. All images were directly called and processed in Google Earth Engine (Figure 2). Google Earth Engine is a cloud-based remote sensing image processing platform developed by Google that can perform online processing and analysis of large-scale remote sensing images. The programming language used is JavaScript, and by referring to the Google Earth Engine help, code examples can be modified for execution, and the final results are presented in the form of output images. The specific image products and parameters for each type are shown in Table 1.

**Table 1:** Remote sensing datasets.

Image Type	Product name	Band name	Spatial resolution
Land surface temperature	MYD11A1.006 Aqua Land Surface Temperature and Emissivity Daily Global 1 km	LST_Day_1 km	1 km
NDVI	MOD13Q1.006 Terra Vegetation Indices 16-Day Global 250 m	NDVI	250 m
NDBI	USGS Landsat 8 Collection 1 Tier 1 and Real-Time data OLI Raw Scenes	B4, B5	30 m
Night light	VIIRS Nighttime Day/Night Band Composites Version 1	avg_rad	500 m

Land Surface Temperature (LST) refers to the temperature of the Earth’s surface as measured from a remote sensing perspective, typically using satellite-based sensors. It represents the heat energy emitted by the land surface, including natural features like soil, vegetation, and water bodies, as well as human-made structures.

Normalized Difference Vegetation Index (NDVI) and Normalized Difference Building Index (NDBI) is remote sensing and image analysis techniques. They are derived from the analysis of multispectral or hyperspectral data captured by sensors on satellite platforms or aircraft. NDVI is a widely used remote sensing index that quantifies the presence and health of vegetation in a given area. It is an essential tool in various fields such as agriculture, forestry, ecology, and environmental science. It enables the monitoring of vegetation growth, health, and distribution over time. Normalized Difference Building Index (NDBI) focuses on quantifying the spectral differences between urban impervious surfaces (like buildings and roads) and natural surfaces (such as vegetation or soil) in the imagery. By comparing the near-infrared (NIR) and shortwave infrared (SWIR) bands of the spectrum, the NDBI highlights areas with high reflectance in the SWIR band and low reflectance in the NIR band, which are typical characteristics of man-made structures like buildings and roads.

Night Light Index (NLI), is an index published by NASA, which calculates the NLI that characterizes changes in human activities by comparing images of nighttime lights projected onto the Earth’s surface from space with images of standard day/night patterns and analyzing the locations where changes occur. The measurement and analysis of night lights have become essential tools for various applications, including urban planning, environmental monitoring, economic analysis, and disaster response.

#### 2.2.4 Other auxiliary datasets

All the administrative boundaries at the scale of 1:4,000,000 maps were obtained from National Earth System Science Data Center,^1^ which we used for the study.

### 2.3 Methods

#### 2.3.1 Co-location analysis methods

**Figure 3:**
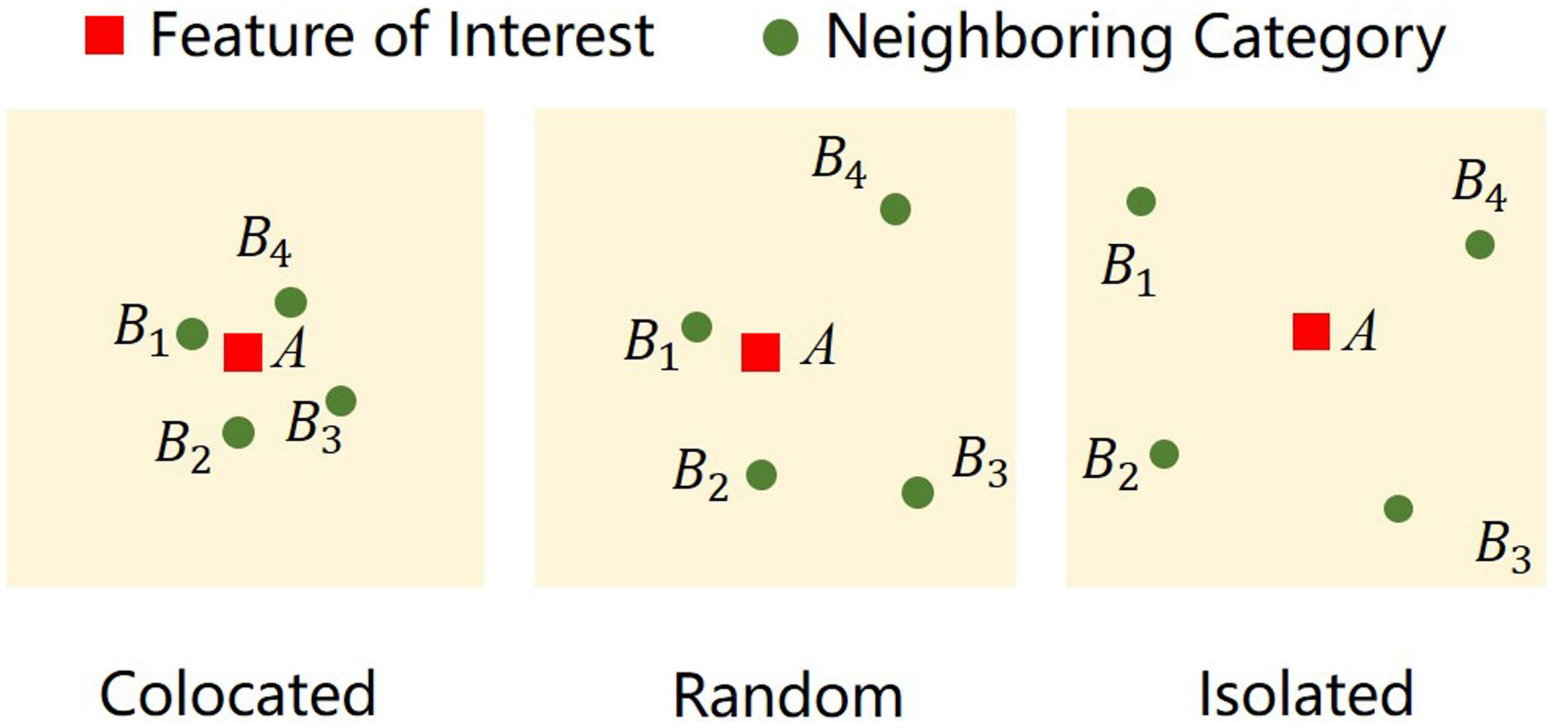
Schematic diagram of the co-location pattern.

The co-location pattern refers to the spatial distribution pattern of an event at different scales, used for the analysis of clustering patterns of point sets in space (24). The co-location pattern analysis method will use the Co-location Quotient (CLQ), which was proposed by Timothy (25). There are two key indicators in this method, the Global Co-location Quotient (GCLQ) and the Local Co-location Quotient (LCLQ). The GCLQ result can reflect the degree to which a feature as a whole affects another feature and can quantify spatial synergies. The LCLQ is an improvement based on the GCLQ, which is used to measure the local scale co-location pattern between two types of point features, explaining the spatial variability between one feature and each feature of another feature class (Figure 3). In recent years, the development of big data technology has improved the efficiency of obtaining massive amounts of data (26). Point of interest (POI) data is increasingly used for urban analysis due to its low cost and high temporal resolution (27–31). Many POI data use categorical variables instead of interval and ratio variables, requiring specific analytical methods to measure the spatial correlation and heterogeneity of categorical point data (32). Compared with other methods of measuring spatial correlation, such as Moran’s I for interval and ratio data, the shared co-location pattern among multiple categories is particularly suitable for urban analysis. Leslie & Kronenfeld developed the GCLQ (25), and Cromley et al. developed the LCLQ by incorporating geographically weighted methods into the GCLQ and optimizing it (33). CLQ is now widely used in urban research, such as job-residence relationships within cities (34), industrial layout characteristics of urban clusters (35), the impact of industrial location patterns on cities (36), and spatial co-location patterns of urban crime (37). The Formula (1) for GCLQ is as follows:


(1) $${\text{GCLQ}}_{A\to B}=\frac{N_{A\to B}/N_{A}}{N_{B}/\left(N-1\right)}\;\;$$

$GCLQ_{A\to B}$ represents the degree to which feature point A is influenced by feature point B; N represents the total number of all feature points in the data set; $N_{A}$ and $N_{B}$ represent the number of feature points A and B, respectively, and $N_{A\to B}$ represents the number of feature points A near B. $N_{A\to B}/N_{A}$ represents the proportion of the number of times feature point A is attracted by feature point B; $N_{B}/\left(N-1\right)$ represents the proportion of feature points B near feature point A. If the value of $GCLQ_{A\to B}$ is greater than 1, it indicates that feature points A and B have spatial correlation; otherwise, if $GCLQ_{A\to B}$ is less than 1, it indicates that feature point B exhibits a scattered (isolated) characteristic in its impact on A in space.

Due to the spatial correlation between two features changing with distance, Cromley proposed the local indicator of the LCLQ to quantify the spatial correlation between two features at a specific distance and visualize it for a more intuitive expression of the spatial relationship between the two features. The Formula (2) for LCLQ is as follows:


(2) $$LCLQ_{A_{i}\to B}=\frac{N_{A_{i}\to B}}{N_{B}/\left(N-1\right)}\;$$

(2-1) $$N_{A_{i}\to B}=\sum_{j=1}^{N}\left(\frac{w_{ij}f_{ij}}{\sum_{j=1\left(j\neq i\right)}^{N}w_{ij}}\right),\left(j\neq 1\right)$$

(2-2) $$w_{ij}=\exp\left(-0.5\times \frac{d_{ij}^{2}}{d_{ib}^{2}}\right)$$

$LCLQ_{A_{i}\to B}$ represents the extent to which feature point B is influenced by point $A_{i}$; In Formula (2-1), $N_{A_{i}\to B}$ represents the weighted average of point B near $A_{i}$; $f_{ij}$ represents whether j belongs to feature point B (1 for yes, 0 for no); $w_{ij}$ represents the weight of j, indicating its importance to $A_{i}$; $d_{ij}$ represents the distance between $A_{i}$ and j; $d_{ib}$ represents the bandwidth distance near $A_{i}$. Similarly, when the value of $LCLQ_{A_{i}\to B}$ is greater than 1, it indicates that feature point $A_{i}$ has a spatial co-location pattern with feature B. If the value of $LCLQ_{A_{i}\to B}$ is less than 1, it indicates that feature point $A_{i}$ has a spatial segregation pattern with feature B. If the value of $LCLQ_{A_{i}\to B}$ is equal to 1, it indicates that feature point $A_{i}$ and feature B have no spatial co-location pattern and are in a state of random distribution. The Formula (2-2) is a Gaussian density function, which produces differentiated effects on each adjacent element B of $A_{i}$ with specified weights, so that the elements B near $A_{i}$ can be marked with bandwidth instead of being limited to the influence of spatial distance.

In this study, COVID-19 confirmed cases are used as element points A, and various types of facility points are used as element points B. This study attempts to use GCLQ and LCLQ values to analyze the spatial correlation features between Wuhan’s COVID-19 confirmed cases and different types of facilities, providing a new perspective for studying the diffusion process and influencing factors of the COVID-19 epidemic within the city. Currently, this method can be calculated using ArcGIS Pro 2.8. In the calculation process, by setting different bandwidth distances, the co-location patterns at different spatial levels can be measured and observed.

#### 2.3.2 COVID-19 risk influencing factor analysis methods

For factors that may have interactive effects, this study used Geodetector for analysis. Geodetector were proposed by Jingfeng Wang in 2010 (38). Geodetector can segment a set of spatial data according to its scale to form spatial heterogeneity features, and test the interaction of multiple variables beyond the correlation of conventional independent variables. Spatial stratified heterogeneity uses the core measurement indicator q value in Geodetector for statistical testing. There are four spatial heterogeneity testing indicators in Geodetector, namely, interaction detector, risk detector, factor detector, and risk detector (39). The core idea is to make a hypothesis for a set of dependent variables: if a independent variable (must be discrete) has a significant impact on this dependent variable (which can be continuous or discrete), then the spatial distribution of the independent variable and dependent variable should be similar. Geodetector are good at analyzing categorical data, but for ordinal, ratio, or interval data, appropriate discretization is required before using Geodetector for statistical analysis. Geodetector default to comparing data one by one. If multiple independent variables are tested with the dependent variable at the same time, the independent variables need to be simply connected to achieve the purpose of jointly testing the dependent variable with multiple independent variables and other independent variables, but the independent variable data must be discrete. Therefore, Geodetector can detect various combinations of numerical data and qualitative data, which is a major advantage of Geodetector (40). Compared with traditional correlation analysis, the biggest advantage of Geodetector is to detect the interaction between two factors and the dependent variable, and the independent variable factors can be either separate or combined. In traditional methods, the general way to identify the interaction is to multiply two groups of independent variables in regression analysis and conduct statistical testing to observe their significance indicators. However, the interaction between many factors may not necessarily be a multiplication relationship. Geodetector precisely make up for this shortcoming. It will traverse all independent variables for double combination, calculate and compare the q values of each single factor and the q value after the two factors are superimposed, and compare the independent q value with the interaction q value to determine whether there is an interaction between the two factors. Based on the results of independent and interaction, the strength, direction, linearity or non-linearity of the interaction can be judged, and all relationships can be output and visualized. The core Formula (3) of the q value test model is as follows:


(3) $$q=1-\frac{\sum_{h=1}^{L}N_{h}\sigma_{h}^{2}}{N\sigma^{2}}=1-\frac{SSW}{SST}$$

(3-1) $$SSW=\sum_{h=1}^{L}N_{h}\sigma_{h}^{2}$$

(3-2) $$SST=N\sigma^{2}$$

In the Formula (3), h represents the discretization or stratification (Strata) of the variable Y or factor X. The numerical labels after stratification only represent inter-group differences, and there is no difference in weight. $N_{h}$ and N are the numbers of units in layer h and the whole region, respectively. $\sigma_{h}^{2}$ and $\sigma^{2}$ are the variances of the stratified Y values in layer h and the whole region, respectively. SSW in Formula (3-1) and SST in Formula (3-2) are the sum of within-group variances and the total variance of the entire region, respectively. The value range of q is [0,1]. The closer q is to 1, the more obvious the spatial heterogeneity of Y is, and the stronger the explanatory power of the independent variable X on attribute Y. If the value of q is equal to 1, it indicates that the factor X completely controls the spatial distribution of Y. If the value of q is 0, it means that there is no relationship between the factor X and Y. Therefore, the value of q represents to what extent the independent variable X explains the dependent variable Y.

This study will conduct an attribution analysis of the COVID-19 pandemic through multiple factors, explore the main influencing mechanisms, and use spatial stratification heterogeneity testing methods to verify the results and quantify the specific modes and degrees of differences. In terms of the correlation between the COVID-19 pandemic and socio-economic attributes, this article will fit continuous independent variables such as linear correlation and curve estimation to identify the main socio-economic influencing factors of the pandemic. For exploring the interaction effects of multiple variables, the q statistic will be used to stratify the independent variables according to certain rules, traverse all possible interaction possibilities, and find the composite influencing factors.

## 3 Result

### 3.1 COVID-19 spatial pattern analysis

**Figure 4:**
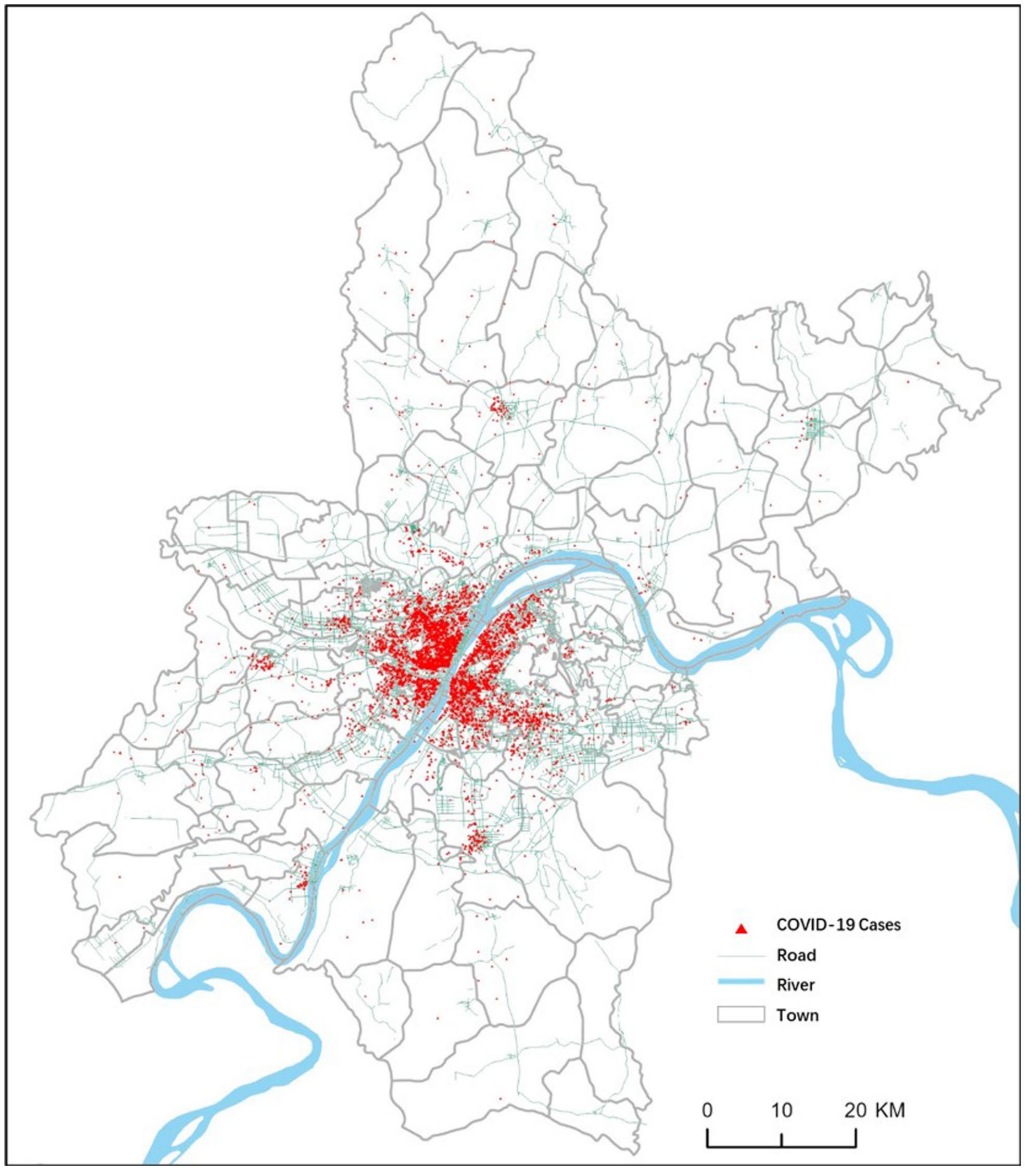
COVID-19 cases in Wuhan from Jan 1th to Jan 22nd, 2020.

Figure 4 shows the spatial location of COVID-19 cases. The epidemic’s spatial risk distribution can be depicted through hotspots formed by the clustering of case points, reflecting the concentration of clustered outbreaks and the level of epidemic risk. Analyzing the spatial pattern of these hotspots can be achieved through kernel density analysis, which categorizes the COVID-19 case clustering distribution into five levels. In Wuhan city, the overall risk distribution of the epidemic appears continuous and clustered in the central urban area, indicating a higher risk level. Conversely, the suburbs display scattered and smaller risk zones with lower risk levels. Moreover, the central urban area on the west bank of the Yangtze River exhibits a higher risk level and larger scale compared to its counterpart on the east bank. Specifically, due to Jianghan District’s high population density and its status as the outbreak center, residents face an extremely high risk of infection, making it the area with the highest spatial risk in Wuhan. The southern part of Jiang’an District also faces heightened risk due to its proximity to Jianghan District and its position as the administrative district with the largest population in Wuhan. While other central urban areas exhibit clustered case distributions, far suburban counties also display risk zones within towns with population concentrations, albeit with weaker spatial continuity and correlation. The spatial distribution pattern of epidemic risk is influenced not only by the population size and density but also by the distance to the outbreak center and spatial adjacency. In Wuhan, the Yangtze River, as a natural feature, runs through the urban, causing spatial variations in transportation accessibility, which impacts the spread of the epidemic.

To explore the global correlation of COVID-19 in Wuhan and its fine-scale spatial heterogeneity, this study will conduct spatial correlation tests on epidemic risk areas in the city. It aims to analyze the spatial distribution patterns of epidemic hotspots and risk based on distance and adjacency at a local scale. The global-scale spatial correlation test employs Moran’s I spatial autocorrelation index. The obtained Moran’s I index is 0.543538, with a z-score of 11.700299. Passing the global test indicates a high degree of spatial correlation between streets with COVID-19 cases and their spatial distance and adjacency, setting the stage for further exploration of hotspots and clustering at a finer scale.

### 3.2 COVID-19 co-location analysis

#### 3.2.1 GCLQ

The use of spatio-temporal scan statistics and spatial neighborhood methods can only describe the overall distribution characteristics of spatial epidemic risk in cities from a global and large-scale perspective of epidemic risk and prevention and control. In fact, the city is a space with a very complex structure and various facilities, which can generate great spatial heterogeneity at a very small scale. Therefore, this article takes various facilities in the city as important variables that affect the spatial heterogeneity of epidemic risk and conducts a refined risk characteristic study. Based on the co-location pattern analysis method, this article analyzes the synergy of epidemic spatial risk for 27 major urban facilities in Wuhan one by one, summarizes and quantifies the impact of different facilities on epidemic spatial risk. In the global synergy location analysis, the results of the Wuhan City 27 urban facilities’ GCLQ for early COVID-19 cases in Wuhan are shown in Table 2.

**Table 2:** GCLQ results.

Category	Name	GCLQ500 m	GCLQ1000m	GCLQ1500 m	GCLQ2000m
Work	Enterprise	0.75	0.83	0.87	0.90
Shopping	Farmers’ market	1.04*	1.00*	0.98*	0.97
	Shopping mall	0.86	0.93	1.01*	1.02*
	Convenience	1.02*	0.96	0.93	0.96
Transportation	Subway station	1.15*	1.16	1.11	1.16
	Bus stop	0.75	0.73	0.72	0.73
	Parking	1.02	1.02	1.03	1.03
	Long-distance bus station	0.53	0.57	0.63	0.65
	Railway station	0.37	0.69	0.77	0.94*
Dining	Chinese restaurant	0.94	0.96	0.98	0.99
	Foreign restaurant	0.75	0.87	0.97	1.01*
	Fast food restaurant	0.87	0.93	0.97	0.99*
	Dessert restaurant	0.95	1.03*	1.07	1.10
Residence	Accommodation	0.79	0.84	0.93	0.95
	Residential building	1.15	1.08	1.05	1.05
	Toilet	0.77	0.81	0.82	0.80
Living	Life service	0.98	0.99	0.99*	1.00*
	ATM	1.05	1.05	1.05	1.06
	Bank	1.13	1.09	1.09	1.10
culture facilities	schools	0.99*	0.89	0.93	0.89
	Library and cultural center	0.95	0.98*	1.00*	1.05
	University	0.62	0.60	0.65	0.67
entertainment	Scenic spots	0.80	0.82	0.84	0.88
	Entertainment	1.05	1.05	1.05	1.06
	Movie theater	0.98	1.01*	1.02	1.02
Medical	Hospital	1.11	1.14	1.17	1.15
	Clinic	1.00*	1.00*	0.97*	0.96

* represents a value of p greater than 0.05 and did not pass the significance test, while the others passed the significance test.

Using the co-location analysis tool in ArcGIS Pro and after multiple experiments, the bandwidth was ultimately set to 500 meters, 1,000 meters, 1,500 meters, and 2000 meters for scanning. Monte Carlo simulation was used for 1,000 iterations to conduct significance tests and obtain the results of the GCLQ (Table 2). Overall, as the distance of the bandwidth increased, the influence of Wuhan’s urban facilities and COVID-19 cases became more and more significant. However, the spatial coordination represented by the GCLQ values of different bandwidth distances was not entirely the same. Based on the results of the GCLQ from Table 2, we categorize the spatial risk of facilities into four co-location types. The first type of co-location pattern facilities in Table 2 is summarized as a sustained collaborative type, such as parking lots, residential areas, ATMs, banks, entertainment venues, and hospitals. The specific feature is that the GCLQ values are all greater than 1, indicating that these facilities have a significant collaborative impact on COVID-19 cases, regardless of short or long distances. The second type of co-location pattern facilities in Table 2 is summarized as a long-distance collaborative type, such as subway stations, dessert restaurants, and movie theaters. The specific feature is that they are not significant in short distances, but the value of GCLQ becomes significant and greater than 1 as the distance increases, indicating that the impact of these facilities on COVID-19 cases gradually increases over a longer distance. The third type of co-location pattern facilities in Table 2 is summarized as a collaborative trend type, such as shopping centers, convenience stores, enterprises, long-distance bus stations, train stations, Chinese restaurants, foreign restaurants, fast food restaurants, accommodations, life services, and libraries/cultural centers and scenic spots. The specific feature is that the GCLQ values are less than 1 in short distances, showing an isolation pattern. However, as the distance increases, the GCLQ value gradually increases, indicating that the impact of these facilities on COVID-19 cases is initially dispersed in short distances, but the dispersed impact weakens and the trend of collaborative impact strengthens as the distance increases. The fourth type of co-location pattern facilities in Table 2 is summarized as a non-collaborative type, such as bus stops, toilet, primary and secondary schools, and colleges. The specific feature is that the GCLQ values are all less than 1, showing an isolation pattern. As the distance increases, the collaborative impact of these facilities on COVID-19 cases does not increase significantly. The fifth type of co-location pattern facilities in Table 2 is summarized as an unknown risk type, such as farmers’ markets and clinics. The specific feature is that the collaborative impact of these facilities on COVID-19 cases is unknown because their GCLQ values were not significant in multiple bandwidth calculations. Based on the above co-location pattern characteristics and the display meaning represented by GCLQ, the risk characteristics were summarized and shown in Table 3.

**Table 3:** Co-location patterns between facility types and COVID-19 risk.

Type of co-location mode	Short-range co-location mode	Long-range co-location mode	Whether there is a co-location trend	Spatial risk level
Continuous collaborative	Collaboration	Collaboration	Yes	+++
Remote collaboration	Insignificant	Collaboration	Yes	++
Collaboration trend	Insignificant	Insignificant	Yes	+
Non-synergistic	Isolation	Isolation	No	−
Unknown risk	Insignificant	Insignificant	No	+++

“+” represents the risk level, and the more of them, the greater the risk.

Due to the existence of collaborative features or collaborative trends in the space of sustained collaborative, long-distance collaborative, and collaborative trend types, in other words, there is a risk of clustered infections in activities carried out in these types of places or in scenarios that involve them. Therefore, there is a certain level of spatial risk, and higher-level health monitoring should be conducted for people and close contacts involved in activities in facilities of this type. Non-collaborative types do not exhibit collaborative features at short or long distances, nor do they exhibit collaborative trends at multiple distances. Therefore, there is no apparent spatial risk for activities carried out in places containing this type, and residents participating in activities in facilities of this type are advised to implement lower-level prevention and control measures. Unknown risk types do not exhibit significant isotopic patterns at multiple distance scales, and the epidemic spatial risk of activities carried out in facilities of this type cannot be simply explained by distance and may be influenced by other factors, resulting in other unknown risks. The uncertainty of such risks is extremely high, so this article determines the spatial risk level of this type as a high-level risk.

According to the results of GCLQ, the multi-distance isomorphic patterns are all shown to be clustered and validated facilities (Parking, Residential, ATM, Bank, Entertainment, Hospital).

#### 3.2.2 LCLQ

**Figure 5:**
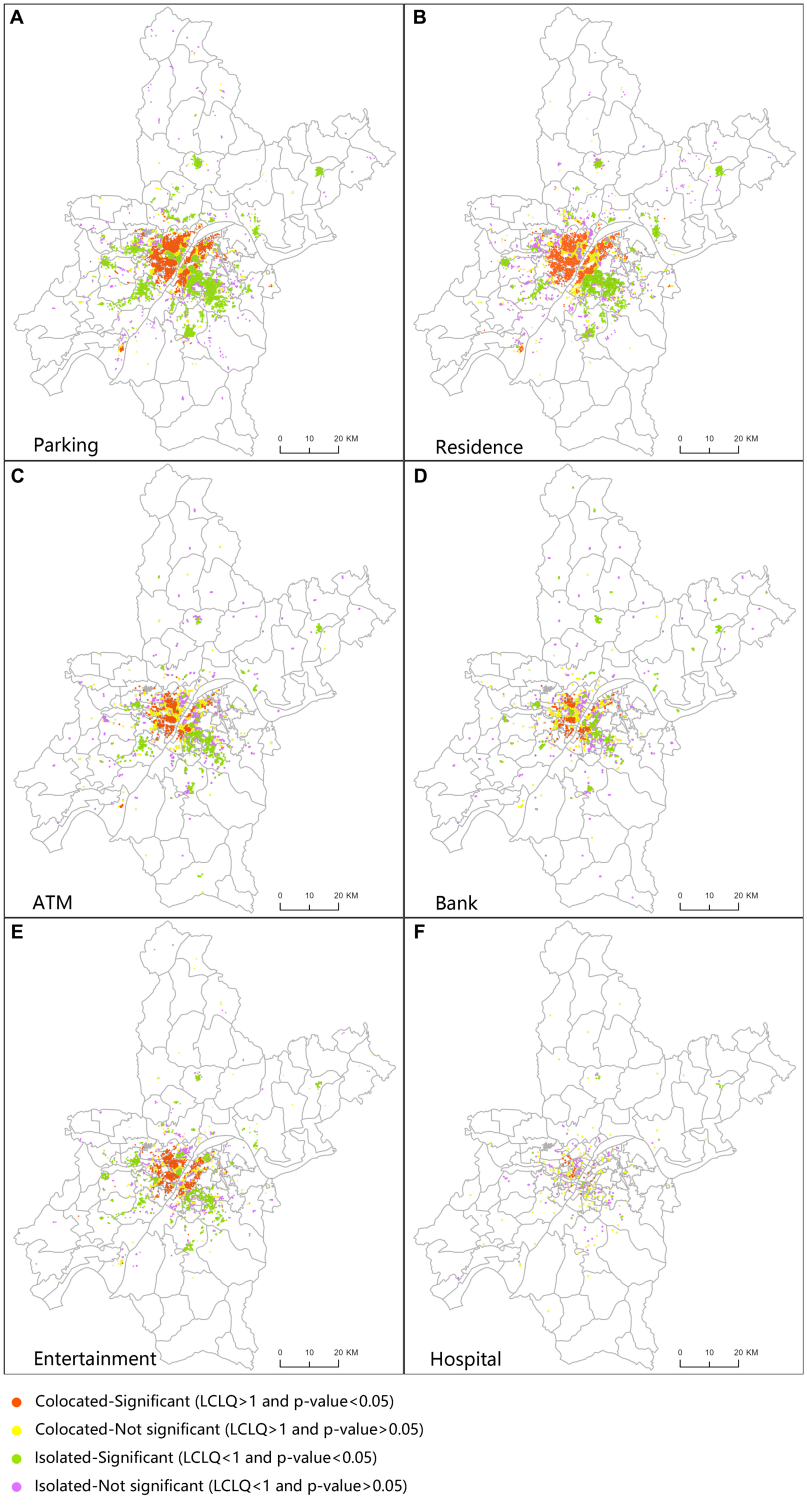
Spatial distribution about urban facilities of local Co-location quotient (LCLQ).

Using the LCLQ for spatial visualization and analysis of results, each facility is discussed one by one, analyzing its co-location with COVID-19 case points in spatial view, and summarizing its facility risk characteristics. When the LCLQ is greater than 1, the larger its value, the more significant the aggregation pattern presented. Conversely, when the LCLQ is less than 1, the smaller its value, the more obvious the “Isolated” co-location pattern presented. Meanwhile, when the value of p tends to 0, there is a high confidence level for the co-location pattern presented by LCLQ. Based on the LCLQ values and value of ps, we categorize co-location patterns into the following four cases: The LCLQ value is greater than 1 and the *p* value is less than or equal to 0.05, indicating a significant “Colocated” co-location pattern. The LCLQ value is greater than 1 and the p value is less than or equal to 0.05, indicating a not-significant “Colocated” co-location pattern. The LCLQ value is greater than 1 and the p value is less than or equal to 0.05, indicating a significant “Isolated” co-location pattern. The LCLQ value is greater than 1 and the p value is less than or equal to 0.05, indicating a not-significant “Isolated” co-location pattern. We show the LCLQs of the facility sites on the map and observe the co-location patterns of each facility type for the COVID-19 case sites (Figure 5).

Based on the spatial distribution characteristics of the LCLQ facilities of the six types of facilities in Figure 5, overall, facilities with a synergistic spatial pattern show a distribution pattern that extends from the outbreak center to the periphery, with strong central synergy and strong peripheral isolation. The Jianghan and Jiang’an districts on the west bank of the Yangtze River have the highest proportion of urban facilities with synergistic street patterns, and many streets in the Wuchang district on the east bank of the Yangtze River also show a large number of synergistic urban facilities. Among the distribution characteristics of the six types of facilities’ spatial patterns, there are also some special phenomena. Specifically, parking lots have a large number of distribution in the north–south direction, but in the complex urban land-use areas of Jianghan District, they have a distribution pattern of isolation. Similarly, banks have a larger number of distribution in the city center with an isolation pattern, often located near residential and office buildings.

### 3.3 COVID-19 risk influencing factor analysis

The risk of the spread and diffusion of the epidemic in space is not only related to spatial adjacency and distance. Since the city is a space where human resources and facilities are highly concentrated, natural and human environments can change greatly over short distances. Therefore, the factors behind the risk of epidemic transmission in the city need to consider the influence of the city’s natural and socio-economic factors, especially the unknown facility risks that cannot be explained by spatial autocorrelation patterns. Attribution needs to be combined with other factors. However, these factors may not necessarily independently affect the spatiotemporal risk characteristics of the epidemic and may produce interaction patterns of influence. Based on the attribution analysis method mentioned, this study comprehensively analyzes the use of the factor detector and interaction detector in the geographical detector.

In this study, the street scale is used as the geographical detection unit, and the road network density, subway stations, hospitals and clinics, and the number of farmers’ markets are counted as independent variables of social environmental elements. At the same time, surface temperature, normalized difference vegetation index (NDVI), normalized difference building index (NDBI), and nighttime light index are used as independent variables of natural environmental elements to test the spatial risk differentiation characteristics of urban factors. This study takes the total number of epidemic cases in each street of Wuhan in the early stage of the epidemic from January 1 to January 22, 2020, as the dependent variable Y, and the above natural and socio-economic elements as well as clustering elements as independent variables X, and conducts spatial differentiation detection in the geographical detector. According to the calculation rules of the geographical detector, this study needs to first discretize the independent variables, and the grading method uses the mean and standard deviation grading method, and the threshold values and calculation methods of each category are shown in Table 4.

**Table 4:** Geodetector variable grading methods.

Classification Methods	Grading annotation
X ≤ −1.5 std. Dev	1
-1.5 std. Dev ≤ X ≤ −0.5 std. Dev	2
-0.5 std. Dev ≤ X ≤ 0.5 std. Dev	3
0.5 std. Dev ≤ X ≤ 1.5 std. Dev	4
X ≥ -1.5 std. Dev	5

The numbers in the classification label only represent the category, not the weight or level.

Discretize the independent variables according to Table 4 and use Geodetector to explore the spatial risk factors of COVID-19. The q value, an important indicator of Geodetector, is one of the important results of spatial heterogeneity, as shown in Table 5.

**Table 5:** Geodetector factor detection results.

Independent variable	Urban factors	*q* value	*p* value
X1	Road density	0.321982	0.000
X2	Subway availability	0.275692	0.000
X3	Number of hospitals	0.402453	0.000
X4	Number of clinics	0.344355	0.000
X5	Farmers’ market	0.426164	0.000
X6	Land surface temperature	0.028092	0.403309
X7	NDVI	0.07778	0.022947
X8	NDBI	0.0634	0.059879
X9	Night lights	0.299839	0.000

*p* value of less than 0.05 indicates significant at the 95% level, and *p* value less than 0.01 indicates significant at the 99% level.

**Figure 6:**
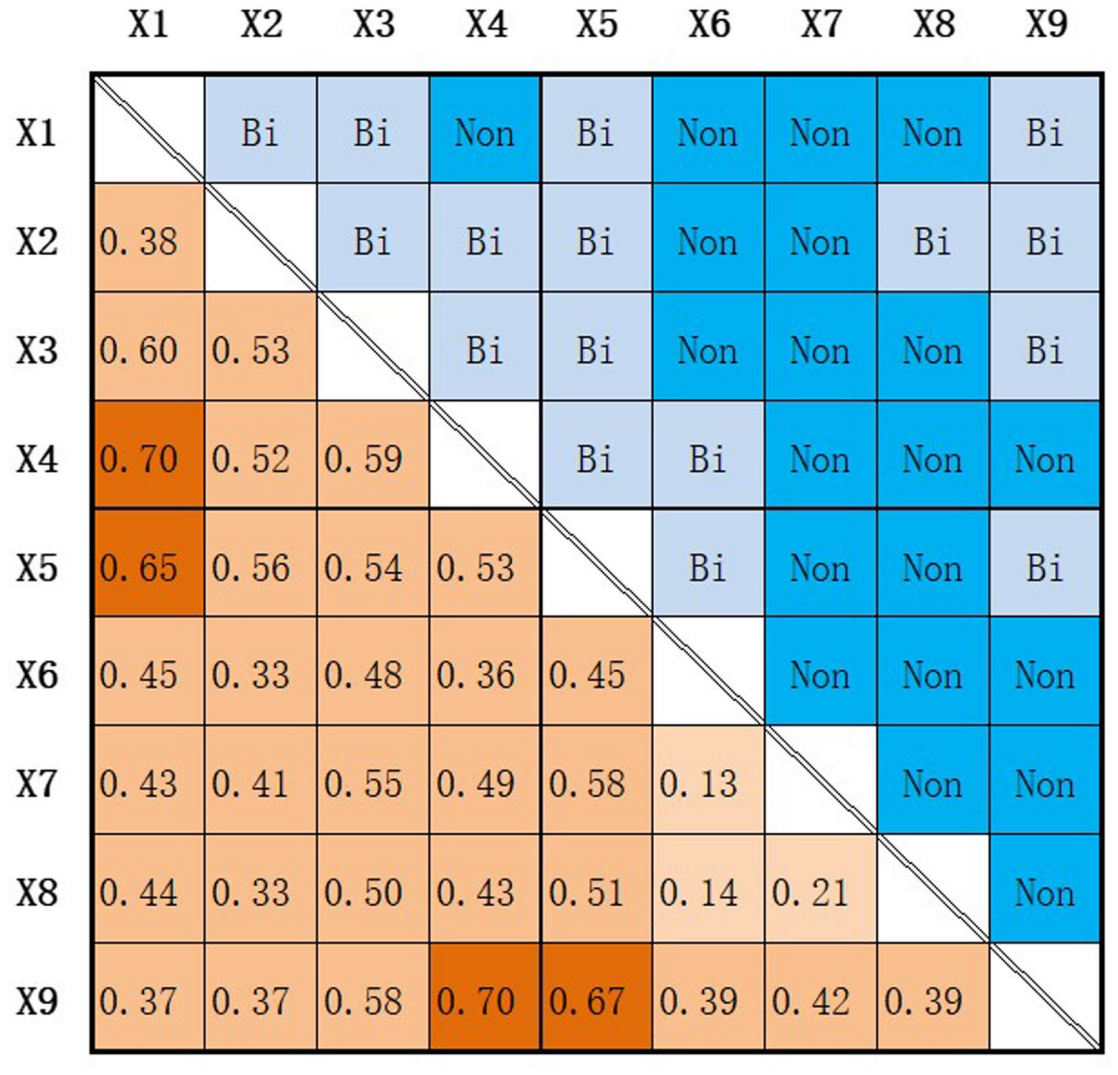
Geodetector Interaction Detection Results. Bi stands for bilinear enhancement, Non stands for non-linear enhancement.

According to the q-values in Table 5, it can be seen that among all the aforementioned urban factors, the number of farmers’ markets, hospitals, and clinics have a certain spatial differentiation in their impact on COVID-19 cases. In other words, the influence of facilities on the spatial transmission risk of COVID-19 varies at different levels in different environmental combinations. To further explore the common factors behind the risk differentiation of the epidemic, the interaction detector in the geographic detector is used for quantitative interpretation. In the interaction detector, when q(X1∩X2) is less than the minimum value of *q(X1)* and *q(X2)*, the explanatory power of *X1* and *X2* on *Y* exhibits a nonlinear decreasing relationship. When q(X1∩X2) is greater than the minimum value of *q(X1)* and *q(X2)*, but less than the maximum value of the two, the explanatory power of *X1* and *X2* on *Y* exhibits a single-factor nonlinear decreasing relationship. When q(X1∩X2) is greater than the maximum value of *q(X1)* and *q(X2)*, the explanatory power of *X1* and X2 on *Y* exhibits a two-factor enhancement relationship. When q(X1∩X2) equals the sum of *q(X1)* and *q(X2)*, the explanatory power of *X1* and *X2* on *Y* are independent of each other. When q(X1∩X2) is greater than the sum of *q(X1)* and *q(X2)*, the explanatory power of *X1* and *X2* on *Y* exhibits a nonlinear enhancement relationship. The results of the interaction detector in this study are shown in Figure 6.

The lower left part of Figure 6 shows the results of the interaction detection between two factors, while the upper right part summarizes the interaction pattern based on the comparison between the interaction detection results and the detection results. The results indicate that all interaction results show enhancement, among which q(X1∩X4)=0.7>q(X1)+q(X4)andq(X4∩X9)=0.7>q(X4)+q(X9), indicating that the spatial risk of clinics for COVID-19 is also influenced by the density of road networks and the functional dense areas with more night lights in cities, possibly because people tend to choose this type of clinics for medical treatment if they are located in dense urban areas or areas with higher road network density, rather than choosing medical facilities that are closer in straight-line distance. In addition, q(X1∩X5)=0.65>q(X1)+q(X5), indicating that the spatial risk of COVID-19 in agricultural markets is also influenced by the density of road networks. Taking the South China Seafood Market as an example, the market is located in the central area of Jianghan District in Wuhan City, and is also located near the Second Ring Road of Wuhan City, which directly connects both sides of the Yangtze River (Erqi Yangtze River Bridge), so the frequent movement of people in this area contributes to the rapid spatial transmission of the epidemic, forming an outbreak center.

The above results explain the unknown spatial risks of epidemic transmission in clinics and agricultural markets in Tables 4, 5 from a multi-factor perspective. From the final results, there are still differences in spatial risks, and the spatial risk of an establishment for people’s epidemic is not only influenced by distance but also requires comprehensive judgment and detection based on urban social and natural environmental factors.

## 4 Discussion

### 4.1 Key findings

This study concentrates on examining the epidemic case points in Wuhan city in conjunction with urban facilities for homotopic pattern analysis. We explore the intricate spatial structure of the city, analyzing the relationships among spatial distances and the distribution of epidemic cases. We explore the spatial correlation of epidemic risk, delving deeper into attributions from the perspective of urban facilities, as well as urban natural and humanistic elements. Our goal is to uncover the diverse characteristics defining the spatial propagation risk of the epidemic across various elements, elucidating the interplay between urban elements and cases. The primary findings of our research are outlined below:

COVID-19 cases show a distinct spatial distribution pattern, spreading from the city center to the periphery. This propagation is influenced by road networks and functional zones, contributing to the varied risk distribution observed across the city. Notably, the central city holds a higher risk level compared to peripheral areas. The Yangtze River acts as a natural barrier, isolating social and economic activities on each side and influencing the spatial risk distribution of the epidemic. Additionally, areas with dense road networks and functional zones exhibiting high nighttime light index correlate with higher case numbers, aligning with findings from previous research. (41) similarly observed that early in the epidemic, confirmed cases were concentrated in Wuhan’s city center before spreading outward.

Although residential addresses sometimes do not coincide with where people become infected with COVID-19, it has been shown that the proximity between these locations remained relatively close within the routines of urban inhabitants (42). Data pertaining to the spatial correlation between infection origins and affected cases frequently lacks comprehensiveness. Spatial data beyond residential addresses is notably less accessible. In instances of an outbreak occurring within an urban facility proximal to residents’ habitats, residential addresses emerge as the most pertinent information. In order to explore more comprehensively the spatial relationship between sources of infection and cases of infection, we use GCLQ and LCLQ analysis revealed a sustained relationship between certain facilities—like parking, residential, ATM, bank, entertainment, and hospital—and COVID-19 case sites. Conversely, the relationship between facilities such as subway station, dessert restaurant, and movie theater and case sites grew as the distance increased. This indicates that these facilities could be significant locations contributing to the concentration and spread of outbreaks. Therefore, these findings imply their potential role as crucial transmission sites.

**Figure 7:**
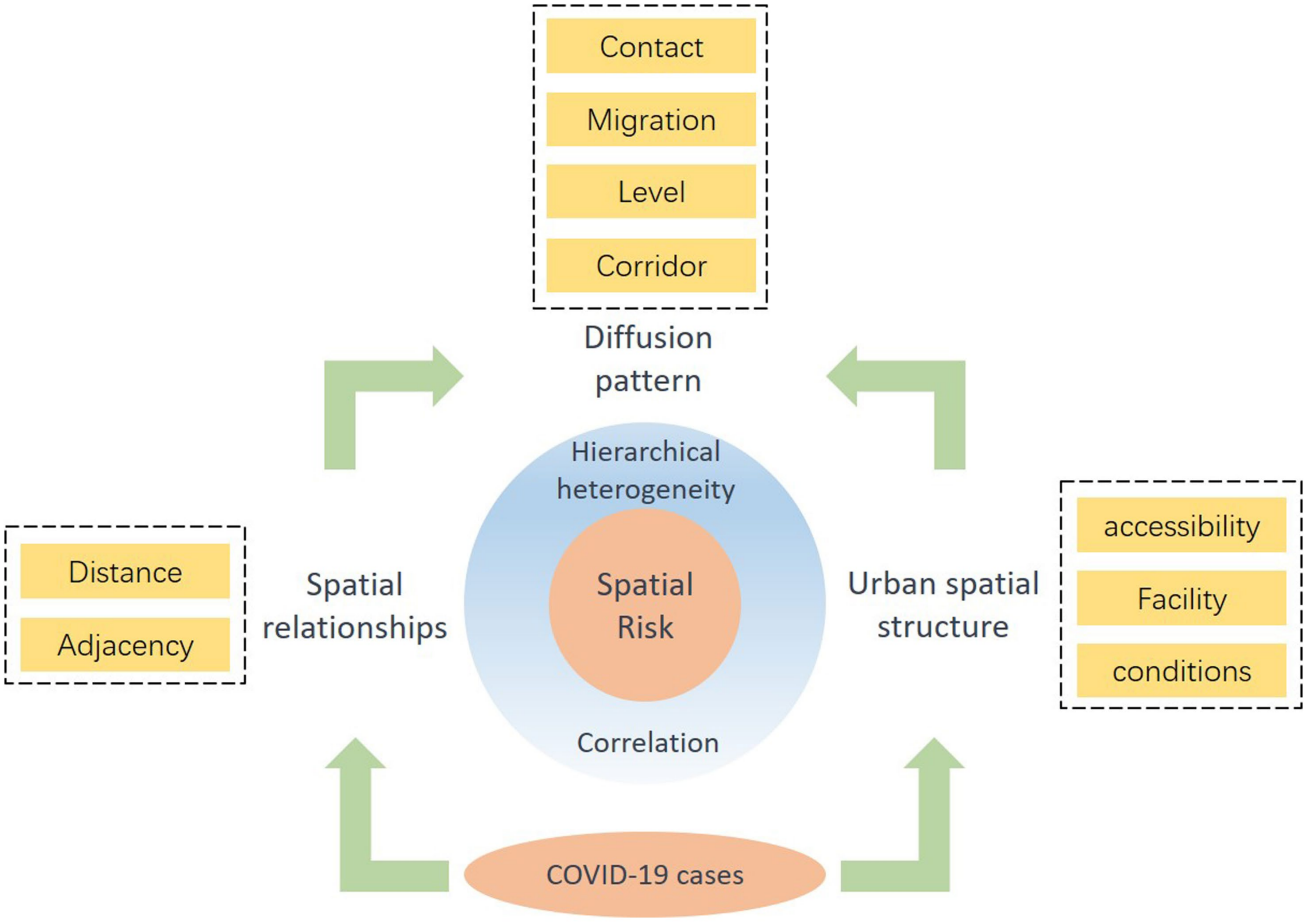
COVID-19 spatial risk relationship.

Geodetector analysis uncovered a significant revelation: the outbreak risk associated with facilities like clinics and farmers’ markets wasn’t solely linked to distance but interacted significantly with various socioeconomic factors (Figure 7). Specifically, the spatial risk of COVID-19 stemming from these facilities wasn’t solely dependent on proximity but demonstrated complex interactions with urban road density and other factors, as scrutinized through Geodetector analysis. For instance, individuals tended to visit clinics situated in densely populated areas or areas with higher road network density, thereby escalating the risk of infection. This study emphasizes how clinics and farmers’ market distinctly impact people’s spatial outbreak risks within the urban social and physical environment. For instance, farmers’ markets, characterized by high foot traffic and confined spaces, pose a higher risk of outbreak transmission compared to clinics. This insight lays the groundwork for tailored and nuanced strategies in epidemic prevention and control.

### 4.2 Research contributions

In terms of academic contributions, this study employs a multifaceted analysis framework centered on urban facilities to quantitatively examine their impact on the spatial risk of COVID-19 outbreaks, thereby enhancing the methodology used in assessing infectious disease risks. The research explores the interconnected mechanism of urban facilities distribution and transportation flow, deepening our understanding of how infectious diseases spread in complex urban settings. Furthermore, the study validates and refines the distance decay law governing infectious disease risk, laying the groundwork for refining theoretical models.

From a practical standpoint, this research offers both theoretical and empirical support for crafting differentiated and scientifically informed COVID-19 epidemic prevention and control strategies. It extends guidance for urban planning and development in the post-epidemic era, fostering the creation of safer, epidemic-resistant urban environments. Moreover, it equips government departments with a reliable quantitative analysis method for more accurate prediction and assessment of infectious disease risks. In this study, the early stage of the first outbreak in Wuhan (January 1 to January 22, 2020) is used as a case study for spatial risk analysis, mainly because people are in a state when they are free to move around completely unaffected by outbreak prevention and control measures, and the results of the study are a more realistic restoration of the COVID-19 transmission process with fewer control variables. The context in which this study was conducted may not occur again in the future, but the results can be used as a scientific reference for the rapid spread of similar infectious diseases in large cities in the future, instead of simply closing down the entire city to minimize losses.

## 5 Conclusion

This study examines the spatial distribution of COVID-19 risk using a research framework based on the spatial co-location model. This model facilitates the implementation of timely hierarchical and segmented control measures to mitigate the risk of infectious disease transmission, even in the absence of a comprehensive understanding of the transmission pattern. Additionally, it aims to forestall unwarranted public panic that might otherwise compel the government to enforce excessively restrictive measures. The analytical outcomes and research insights presented herein can serve as valuable references for decision-making authorities, aiding in the formulation of targeted prevention and control strategies. For facilities with a high risk of infection but lacking statistical confidence in probability results, we use geo-detectors. This approach involves an interaction impact analysis that integrates natural and socio-economic factors to unveil the epidemic’s spatial spread within urban areas. Though future infectious diseases may differ in transmission characteristics and influencing factors from those found in this study, the proposed ideas provide a quick method to identify urban risk facilities and primary influencing factors. Consequently, this study provides a swift method for grading urban risk elements, offering a scientific foundation for the government to implement differentiated measures in response to emerging infectious threats.

Inevitably, this study has some limitations. First, a major limitation of this study is the data acquisition. The selected case points, which represent home addresses, might not cover all transmission locations, thus incompletely reflecting infection risk. Early in an epidemic, the medical community confronts various constraints: insufficient case data, limited testing resources, time pressures due to rapid spread, and incomplete understanding of the virus and its transmission. These factors collectively impede comprehensive epidemiological investigations. While this limitation poses a challenge to pinpointing infection times and places, it offers a unique opportunity for geography to address these gaps through spatial analysis, significantly supporting outbreak responses. Our use of patients’ home locations as case sites captures general areas of case concentration, complemented by urban facility data to assess spatial risk associations. However, future efforts could aim to connect cases to specific public exposure sites where feasible. Second, it’s essential to highlight that this study solely examines early outbreak data in Wuhan, suggesting the need for future research tracking outbreaks over time to understand how various facilities contribute to outbreak risks. It’s also essential to gather outbreak and urban data from other cities for comparative studies. Lastly, developing quantitative models to forecast the impact of facility distribution changes on outbreak risks can offer insights for urban planning.

## Data availability statement

The original contributions presented in the study are included in the article/supplementary material, further inquiries can be directed to the corresponding author.

## Author contributions

GZ: Methodology, Writing – original draft, Writing – review & editing. BM: Conceptualization, Formal analysis, Supervision, Writing – original draft, Writing – review & editing. HL: Project administration, Resources, Software, Writing – review & editing. XZ: Investigation, Project administration, Resources, Writing – review & editing. MX: Data curation, Writing – review & editing. SC: Validation, Writing – original draft. JW: Writing – review & editing, Supervision, Validation.

## Footnotes

^1^ http://www.geodata.cn/
